# On the Scavenging Ability of Scutellarein against the OOH Radical in Water and Lipid-like Environments: A Theoretical Study

**DOI:** 10.3390/antiox11020224

**Published:** 2022-01-25

**Authors:** Maciej Spiegel, Tiziana Marino, Mario Prejanò, Nino Russo

**Affiliations:** 1 Department of Pharmacognosy and Herbal Medicines, Wroclaw Medical University, Borowska 211A, 50-556 Wroclaw, Poland; maciej.spiegel@student.umed.wroc.pl; 2 Dipartimento di Chimica e Tecnologie Chimiche, Università della Calabria, I-87136 Rende, CS, Italy; tmarino@unical.it (T.M.); mario.prejano@unical.it (M.P.)

**Keywords:** antioxidant, reaction mechanism, scavenging power, kinetic constants, hydroperoxyl radical

## Abstract

The antioxidant capability of scutellarein, a flavonoid extracted from different plants of the Scutellaria family, was computationally predicted by considering its reaction with the OOH radical in both lipid-like and water environments. The pKa and equilibrium behavior in the aqueous phase were also calculated. Different reaction mechanisms involving the most populated species were considered. The work was performed by using the density functional level of theory. The individual, total, and fraction-corrected total rate constants were obtained. The results show that scutellarein has scavenging power against the hydroperoxyl radical similar to that of Trolox, which is generally used as a reference antioxidant.

## 1. Introduction

The millenary tradition of Chinese medicine today represents an incentive to the search for natural drugs arising from medicinal plants [1,2,3]. A particular class of natural products of plant origin is the so-called phytophenols, a large group of low molecular weight substances, in which one or more phenolic -OH functional groups may be present [4,5,6]. Among them, the most studied systems are the flavonoids, characterized by a carbon skeleton (C_6_-C_3_-C_6_), in which the C_6_ components are aromatic rings, and C_3_ is a heterocyclic group [7,8]. A large series of these compounds, extracted from a variety of plants and fruits, show interesting antioxidant properties and therefore were proposed as effective drugs against many diseases related to human oxidative stress [8,9,10]. The flavonoid scutellarein (5, 6, 7, 4′-tetrahydroxyflavone) is found in *Scutellaria baicalensis*, *Scutellaria lateriflora*, *Scutellaria barbata*, *Scutellaria baicalensis Georgi* and *Vitis vinifera*, which grow naturally in many East Asian countries, the Russian Federation and some European countries. Recent studies indicate that scutellarein possesses a wide spectrum of pharmacological effects, such as hepatoprotection [11], anti-tumor [12,13], anti-bacterial and anti-viral [14,15], antioxidant [16] activities and efficient activities towards neurodegenerative diseases [17,18]. The various mechanisms of action for the different diseases reported above are still not discovered, but the antioxidant activity is closely linked to the scavenging capacity against oxygen-containing radicals (ROS) like OH, OOH, O_2_^•−^ produced in the human body through Fenton reactions. The main reaction mechanisms by which the antioxidant system (indicated here as H_4_A) can inhibit ROS were established in a series of previous studies [6,8,19,20] and can take place through redox-related pathways:

(a) Hydrogen Atom Transfer (HAT)
  H_n_A + R^•^ → H_n−1_A^•^ + RH
(b) Single Electron Transfer (SET)
  H_n_A + R^•^ → H_n_A^+•^ + R^−^

Or a non-redox related pathway:

(c) Radical Adduct Formation (RAF)
  H_n_A + R^•^ → [H_n−1_A-RH]^•^

In this work, we report a detailed theoretical investigation, performed in the framework of density functional theory (DFT), on the scavenging ability of scutellarein against the OOH radical in both water and lipid-like environments. In the water solution, we have considered all the species, neutral and charged, generated by scutellarein in the physiological pH conditions (7.4).

## 2. Materials and Methods

All the electronic calculations were performed by using the Gaussian 09 Code [21]. Geometry optimizations were performed at the DFT level by using the M06-2X functional [22], coupled with the 6-311 + G(d,p) basis set. Solvation model density (SMD) [23] were employed to simulate water (ε = 78.4) and lipid-like (choosing ε = 4.7 of the pentylethanoate) environments. Frequency computations were done at the same level of theory to establish the stationary point nature (with no imaginary frequency for the minimum and one imaginary frequency for the transition state). The unrestricted procedure was applied for open-shell systems. Intrinsic reaction coordinate (IRC) calculations were performed to verify if the intercepted TS is properly connected to the relative minima (reactant and product) in the minimum energy reaction path. The employed computational strategy was proven to be effective for the computation of thermochemistry, kinetics, noncovalent interactions, and for estimation of pK_a_ values in a series of reaction mechanisms involving several antioxidant molecules [24,25,26,27,28].

Ionization energies (IP), proton affinities (PA), bond dissociation (BDE), and proton desorption (PDE) energies as global reactivity indexes were estimated in the framework of adiabatic approximation.

In particular, the following reactions were considered:

BDE = ∆H (Cx-O) + ∆H (H) − ∆H (Cx-OH); IP = ∆H (4-OH^+^) + ∆H (e^−^) − ∆H (Cx-OH); PA = ∆H (Cx-O^−^) + ∆H (H^+^) − ∆H (Cx-OH); PDE = ∆H (Cx-O) + ∆H (H^+^) − ∆H (Cx-OH^+^)

The solvation enthalpies ∆H (H^+^) (1055.7 kJ/mol) and ∆H (e^−^) (77.5 kJ/mol) were taken from a recent study by Markovic et al. [29].

Relative energies were computed with respect to the sum of separate reactants, and thermodynamics corrections at 298.15 K were taken into account.

The pK_a_ and molar fraction for neutral vs. charged species were obtained by using the previously proposed protocol largely used in antioxidant reaction mechanism studies [30,31].

Rate constants, *k*, were calculated according to the conventional transition state theory [32]. For the mechanism involving SETs, the barriers of reaction were computed using the Marcus theory [33]. For rate constants close to the diffusion limit, the Collins–Kimball theory [34] was applied. More detailed descriptions can be found in the references [35,36].

MarvinSketch version 21.15.0, ChemAxon was used to visualize the structures.

## 3. Results and Discussion

### 3.1. Chemical Equilibria in Water

**Figure 1:**
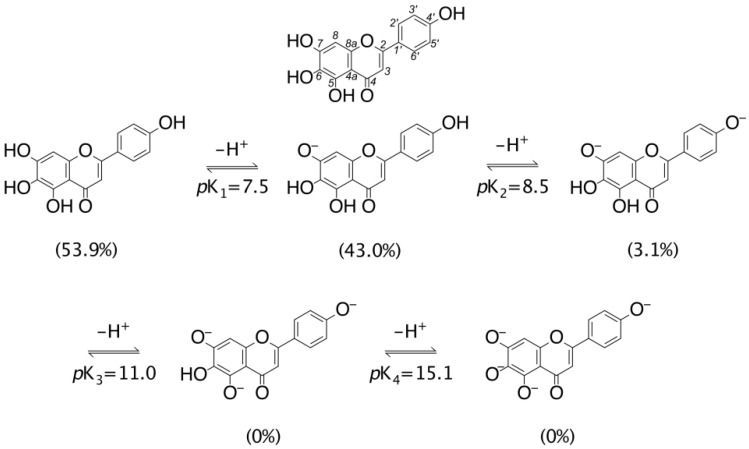
Structure of scutellarein (H_4_A) and pKa values of the relative deprotonation paths and molar fractions of the different scutellarein acid-base species (in parentheses), at physiological pH.

Because of the presence of four OH groups in the scutellarein (hereafter denoted as H_4_A) structure (see Figure 1) and the lack of detailed experimental information, as a first step of the work, we computed the pK_a_ for the possible equilibria in the water solution. The results, depicted in Figure 1, show that the lower pK_a_ can be associated with the deprotonation of the OH group in position C_7_ with a value of 7.50. The second deprotonation step involves the OH group in position C_4′_ of the phenyl ring (pK_a_ = 8.53). The other equilibria were characterized by pK_a_ values of 10.96 (position C_5_) and 15.12 (position C_6_). From the molar fraction as a function of the pH (see Supplementary Materials Figure S1), we found that in the physiological conditions (pH = 7.4), the molar fraction of the neutral species (H_4_A) represented 53.88% of the water solution composition followed by the anionic species H_3_A^−^ and H_2_A^2^^−^ with 42.77% and 3.16%, respectively. The HA^3^^−^ and A^4^^−^ forms were not present at the physiological pH (see Figure 1). For this reason, our study in the water solvent only took into account the most populated H_4_A and H_3_A^−^ species.

Although OOH in physiological conditions is mainly present in its dissociated species, in order to compare our results with those of other antioxidants studied with the same methodology, we considered it in its neutral form.

### 3.2. Reactivity Indices

The computed values indicate lower BDE for the detachment of the hydrogen from the C_6_OH position (Table 1). This result should indicate that the preferred HAT mechanism must involve this OH topology. The obtained adiabatic IP values were sensibly higher than the BDE and slightly decreased in going from pentylethanoate to water solutions (125.3 vs. 112.5 kcal/mol). 

The comparison between the two dominant species present in the aqueous environment clearly showed that the IP for the deprotonated form was smaller by approximately 19 kcal/mol with respect to the neutral one.

The obtained BDE and IP values indicated that for all species and environments considered, the electron transfer reactions should be energetically more expensive than the HAT values.

The PDE result underlines that the preferred site was always the C_6_OH with values ranging from 7.9 (H_4_A in pentylethanoate) to 1.9 (H_4_A in water) and 14.6 (H_3_A^−^ in water) kcal/mol; a different behavior was found for the PA. In fact, the lowest values were found for the C_4′_ position in the case of neutral species in a lipid-like environment and for the mono-deprotonated one in water and C_7_ for H_4_A in water.

### 3.3. Reactions in Lipid-like Environment

**Figure 2:**
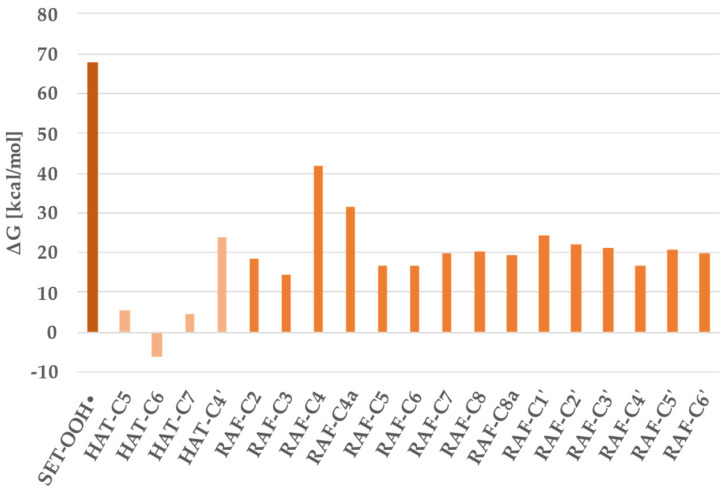
Gibbs reaction free energies of (ΔG) at 298.15 K for scutellarein against hydroperoxyl radical in pentyl ethanoate solvent.

As previously mentioned, and following a well-consolidated approach from the literature [26], we used the pentylethanoate solvent (PE) as a representative of the lipid-like medium. Results for OOH^•^ radicals considering the HAT, RAF and SET reaction mechanisms are reported in Figure 2 and Table 2.

From Figure 2, it emerges that the SET reaction channel is forbidden given the very high Gibbs energy value. Paths involving the RAF also had positive reaction energy values, and their feasibility did not seem possible. The HAT process gave an exergonic value (6.0 kcal/mol) for the proton transfer from the OH group in position C_6_. Smaller endergonic values were obtained for the other H transfer in positions C_5_ (5.7 kcal/mol) and C_7_ (4.6 kcal/mol).

The search of the transition state for the reaction of scutellarein against hydroperoxyl radical was only done for the exergonic and endergonic processes with Gibbs energies lower than 10 kcal/mol, as reported in Table 2.

**Figure 3:**
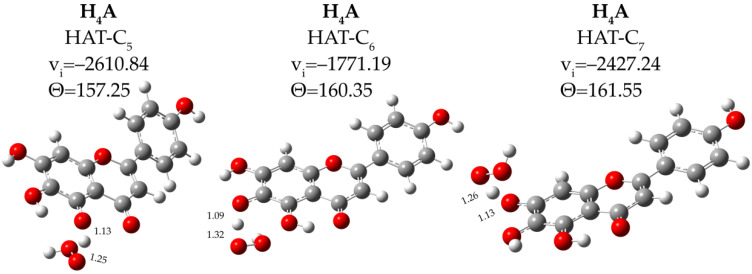
Optimized TS structures with the main geometrical features for the scutellarein in pentylethanoate solvent. Bond lengths in Å, angles (Θ) in degrees and imaginary frequencies (ν) in cm^−1^.

We characterized the transition state (TS) structures for the three considered reactions. The analysis of the computed imaginary frequency (one negative frequency characterizes a TS structure) and relative IRC showed how these structures of TS properly connect reactants and products. Figure 3 shows the TS structures for the considered reactions. The smaller energy barrier (17.0 kcal/mol) occurred for the OOH attack to the C_6_ hydroxyl group, followed by that in C_7_ (23.7 kcal/mol) and C_5_ (28.5 kcal/mol).

### 3.4. Reactions in Water Solution

**Figure 4:**
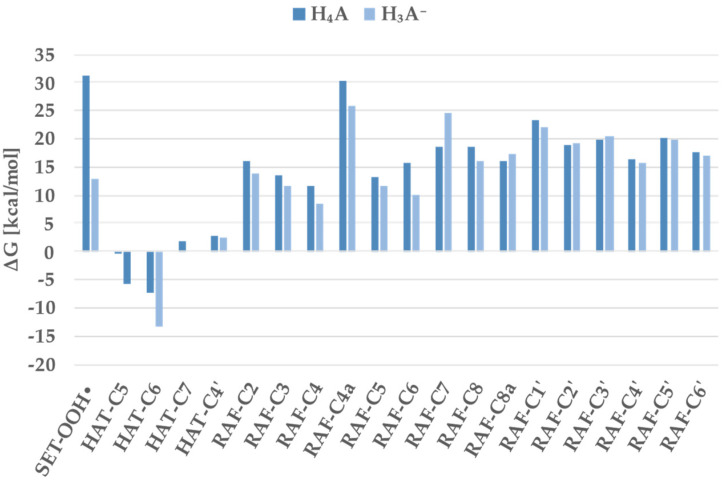
Gibbs free reaction energies (ΔG) at 298.15 K for scutellarein against hydroperoxyl radicals in a water solvent.

Results reported in Figure 4 and Table 3 show that the only negative Gibbs energies occurred for the HAT mechanism involving the C_5_OH and C_6_OH groups for both the neutral and deprotonated forms. In particular, the higher exergonic value is related to the deprotonation of the C_6_OH for both H_4_A (−7.3 kcal/mol) and H_3_A^−^ (−13.2 kcal/mol). Other reactions with positive values within 10 kcal/mol were found and considered in the subsequent kinetic investigation. In detail, these concern the SET and RAF mechanisms for the deprotonated form, the HAT mechanism involving the C_7_OH group in H_4_A, and the one involving the C_4__′_OH group for both the considered species.

**Figure 5:**
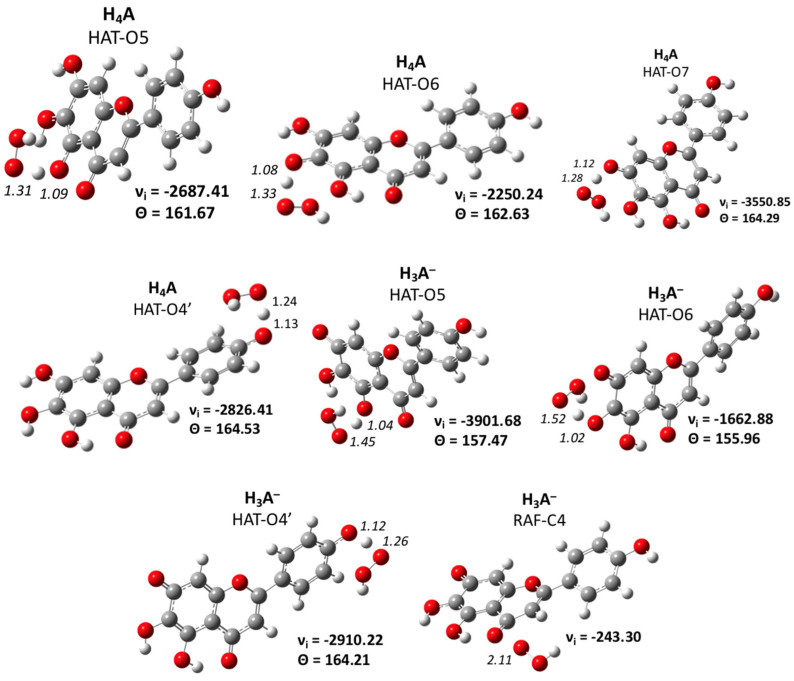
Optimized TS structures with the main geometrical parameters for the scutellarein in a water solvent. Bond lengths are in Å, angles (Θ) in degrees and imaginary frequencies (ν) in cm^−1^.

The corresponding transition states for all the aforementioned processes were found and characterized (see Figure 5). Moreover, in this case, the analysis of the geometries of the negative vibration frequency and the IRC trend confirmed the reliability of the optimized structures.

The lowest energy barriers were found for HAT paths that involve the hydrogen of the group C_6_ OH for both the neutral and anionic species. The energetic cost for the H transfer from the C_5_OH was 22.7 kcal/mol for H_4_A and 20.0 kcal/mol for H_3_A^−^. The highest barrier concerned the RAF mechanism on the C_4_ atom (25.0 kcal/mol).

### 3.5. Kinetic Behavior

The computed rate constants (*k*) and branching ratios (Γ) for the selected paths are given in Table 4. In the lipid-like environment, the dominant channel (99.99%) results were relative to the HAT mechanism on C_6_OH with a *k* value of 1.06 × 10^3^ (M^−1^ s^−1^). The same behavior was found in the water solvent in which a higher *k* was calculated for the H_3_A^−^ form (5.15 × 10^5^ M^−1^ s^−1^). Considering the molar fraction (Table 5), the corrected total rate (*fk*_total_) became 3.80 × 10^3^ (M^−1^ s^−1^) and 2.23 × 10^5^ (M^−1^ s^−1^) for H_4_A and H_3_A^−^, respectively. This result further underlines the importance of considering, in the water phase, the most populated species as resulted by the equilibrium constant prediction.

To have an indication of the scavenging power activity of scutellarein against the hydroperoxyl radical, we compared our results with those of other antioxidants. First of all, the comparison can be validated with Trolox, which is commonly used as a reference antioxidant [35,36]. The rate coefficients for the reactions between Trolox and OOH in nonpolar media (mimicking the lipid-like environment) and in aqueous solution (pH = 7.4) were 3.40 × 10^3^ and 8.96 × 10^4^ M^−1^ s^−1^, respectively [36]. Therefore, scutellarein is predicted to react with the hydroperoxyl radical in a similar manner to Trolox in both nonpolar and aqueous solutions. Furthermore, a *k* comparison with that of chrysin and quercetin [27], structurally similar to our studied system, revealed that in lipid-like media, the scavenging activity of our system (1.06 × 10^3^ M^−1^ s^−1^) was comparable with that of quercetin (4.39 × 10^3^ M^−1^ s^−^^1^) while chrysin was essentially inactive [27].

The performance of scutellarein in the water solution with respect to quercetin was less efficient to inactivate the OOH radical; their kinetic constants were 7.06 × 10^3^ and 8.11 × 10^9^ M^−1^ s^−1^ [27].

Similar trends were obtained if the comparison was made considering the experimental values in aqueous solution and at pH = 7.4, which were 4.1 × 10^5^ and 1.6 × 10^5^ M^−1^ s^−1^ for Trolox and quercetin, respectively [37,38].

## 4. Conclusions

The scavenging abilities of scutellarein against the hydroperoxyl radical in water and lipid-like environments were investigated at a theoretical level by employing the density functional method.

Different reaction mechanisms (HAT, SET and RAF), molecular descriptors (IP, PA, BDE, PDE), pKa and the molar fraction in water solution at physiological conditions (pH = 7.4) were considered.

The results can be summarized as follows:

- The pKa, for the different deprotonation steps, are predicted to be 7.50, 8.53, 10.96 and 15.12. The preferred deprotonation site involves the OH group in position C_6_. At physiological pH, the most populated species are the neutral (H_4_A, 53.9%) and mono-anion (H_3_A^−^, 43.0%) species;
- In the lipid-like environment, the preferred HAT mechanism involving the C_6_OH group, with a k value of 1.06 × 10^3^ (M^−1^ s^−1^), is dominant. The same behavior is found in water in which the higher kinetic constant (k = 5.15 × 10^5^ M^−1^ s^−1^) arises from the H_3_A^−^ form;
- Comparison with Trolox indicates as scutellarein reacts with the hydroperoxyl radical with approximately the same efficiency in both nonpolar and aqueous solutions.

## Figures and Tables

**Table 1:** Computed BDE, IP, PDE and PA for neutral and charged species in water and pentylethanoate solvents. All values are in kcal/mol.

Species	Solvent	OH Position	BDE	IP	PDE	PA
H_4_A	Pentylethanoate	C_5_	82.6	125.3	19.6	61.8
		C_6_	70.9		7.9	59.2
		C_7_	81.5		18.5	56.8
		C_4′_	100.8		37.8	53.6
H_4_A	Water	C_5_	79.7	112.5	8.8	33.3
		C_6_	72.9		1.9	31.7
		C_7_	82.1		11.2	29.8
		C_4′_	82.9		12.0	31.4
H_3_A^−^	Water	C_5_	74.5	93.9	22.1	39.9
		C_6_	67.0		14.6	41.4
		C_4′_	82.6		30.2	33.0

**Table 2:** Gibbs free energy of reaction (ΔG) and activation (ΔG ^‡^) [kcal/mol] at 298.15 K for the HAT mechanisms in a lipid-like environment (the superscript (PE) indicates pentylethanoate solvent).

OH Position	H_4_A^PE^	
	ΔG	ΔG ^‡^
C_5_	5.7	28.5
C_6_	−6.0	17.0
C_7_	4.6	23.7

**Table 3:** Gibbs free reaction energy (ΔG) and activation (ΔG ^‡^) in kcal/mol at 298.15 K for the considered mechanisms in a water environment.

Mechanism	H_4_A		H_3_A^−^	
	ΔG	ΔG ^‡^	ΔG	ΔG ^‡^
HAT (C_5_OH)	−0.5	22.7	−5.7	20.0
HAT (C_6_OH)	−7.3	16.4	−13.2	12.2
HAT (C_7_OH)	1.9	22.7		
HAT (C_4′_OH)	2.7	23.3	2.4	22.7
RAF (C_4_)			8.6	25.0

**Table 4:** Rate constants (*k*) and branching ratios (Γ) of reaction between scutellarein and OOH radical calculated at 298.15 K. *k*_total_ was calculated as the sum of the individual rate constants from the considered reaction paths, while *k*_overall_ is the sum of the rate constants for the different species present in solution at pH = 7.4.

Mechanism	*k* (M^−1^ s^−1^)	Γ (%)	*k* (M^−1^ s^−1^)	Γ (%)	*k* (M^−1^ s^−1^)	Γ (%)
	Pentylethenoate		Water			
	H_4_A		H_4_A		H_3_A^−^	
HAT (C_5_-OH)	4.40 × 10^−4^	0.00	1.04 × 10^1^	0.15	3.92 × 10^3^	0.76
HAT (C_6_-OH)	1.06 × 10^3^	99.99	7.03 × 10^3^	99.55	5.15 × 10^5^	99.24
HAT (C_7_-OH)	1.10 × 10^−1^	0.01	2.01 × 10^1^	0.28		
HAT(C_4′_-OH)	-	-	1.41 × 10^0^	0.02	4.42 × 10^0^	0.00
RAF-(C_4_)					7.62 × 10^−5^	0.00
*k*_total_	1.06 × 10^3^		7.06 × 10^3^		5.19 × 10^5^	
*k*_overall_	1.06 × 10^3^		3.80 × 10^3^		2.23 × 10^5^	

**Table 5:** Molar Fractions (*f*), total rate constant (*k*_tot_ in M^−1^ s^−1^) and fraction corrected total rate constant (*fk*_tot_ in M^−1^ s^−1^), at 298.15 K in aqueous solution at pH = 7.4, for the reaction between scutellarein species and OOH.

Species	*f*	*k*_tot_	*f* *k*_tot_
H_4_A	0.5388	7.06 × 10^3^	3.80 × 10^3^
H_3_A^−^	0.4297	5.19 × 10^5^	2.23 × 10^5^

## Data Availability

Data is contained within the article and supplementary material.

## Supplementary Materials

The following are available online at https://www.mdpi.com/article/10.3390/antiox11020224/s1, Figure S1: Distribution diagram of scutellarein as a function of pH.
