# Supplementary material for: On the Scavenging Ability of Scutellarein against the OOH Radical in Water and Lipid-like Environments: A Theoretical Study
Source: Antioxidants (Basel). 2022 Jan 25;11(2):224. doi: 10.3390/antiox11020224 (PMC8868326; doi:10.3390/antiox11020224)
Supplement: Supplementary file 1 [file antioxidants-11-00224-s001.zip › antioxidants-1543104-supplementary.pdf]

Supplementary information

On the scavenging ability of scutellarein against the OOH radical in water and lipid-like environments. A quantum chemical and kinetic investigation

Maciej Spiegel[1,2], Tiziana Marino[2], Mario Prejanò[2] and Nino Russo[2]

[1] Department of Pharmacognosy and Herbal Medicines, Wroclaw Medical University, Borowska 211A, 50-556 Wroclaw, Poland

[2] Dipartimento di Chimica e Tecnologie Chimiche, Università della Calabria, I-87136 Rende (CS), Italy

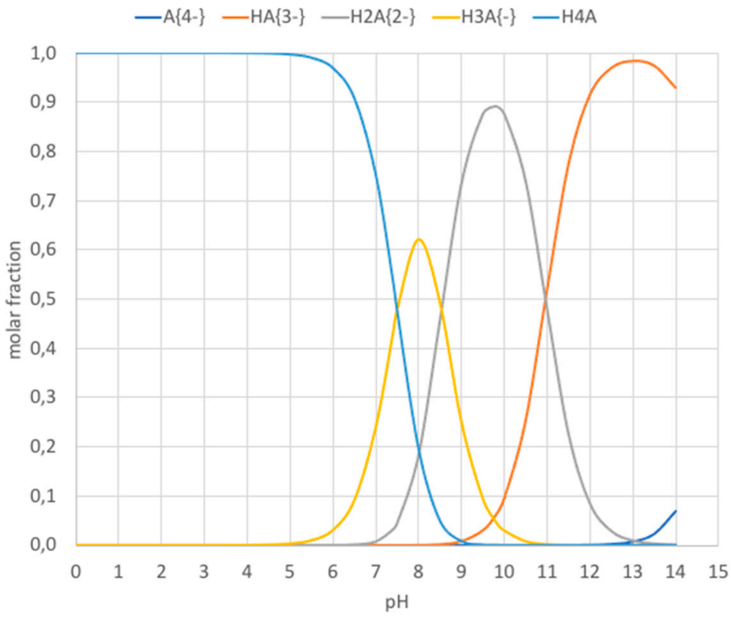

Figure S1. Distribution diagram of scutellarein as a function of pH.
